# *CYP19A1* gene expression in the peripheral blood of Brazilian women with breast cancer relapse

**DOI:** 10.1186/s12885-020-06978-z

**Published:** 2020-05-27

**Authors:** Maria da Conceição Barros-Oliveira, Danylo Rafhael Costa-Silva, Larysse Cardoso Campos-Verdes, Renato de Oliveira Pereira, Rozirene Araújo Silva, Paulo de Tarso Moura-Borges, Emerson Brandão Sousa, André Luiz Pinho-Sobral, Pedro Vitor Lopes-Costa, Alesse Ribeiro dos Santos, Ione Maria Ribeiro Soares-Lopes, Jackeline Lopes Viana, Mariella de Almeida Melo, Fidelis Manes Neto, Eid Gonçalves Coelho, Maria do Socorro Pires e Cruz, Vladimir Costa-Silva, Luiz Henrique Gebrim, Benedito Borges Da Silva

**Affiliations:** 1 Postgraduate Program of the Northeast Network of Biotechnology (RENORBIO), Teresina, Northeast Brazil; 2 Federal University of Piaui, Teresina, Piaui Brazil; 3 Mastology Unit, Getulio Vargas Hospital, Teresina, Piaui Brazil; 4 Perola Byington Hospital, São Paulo, São Paulo Brazil

**Keywords:** Breast cancer, *CYP19A1*, Gene expression, Relapse

## Abstract

**Background:**

The *CYP19A1* gene, which encodes the enzyme responsible for androgen aromatization into estrogens, may play an important role in breast cancer aggressiveness. However, no study has evaluated *CYP19A1* gene expression in the peripheral blood of women with relapsed breast cancer.

**Methods:**

In this cross-sectional study, *CYP19A1* gene expression was quantified by RT-PCR in the peripheral blood of 146 women with breast cancer who were first divided into two groups according to the expression of *CYP19A1* (low and high); each group had 73 patients. Subsequently, women were divided into two groups: those without recurrence (control, *n* = 85) and those with recurrence (study, *n* = 61). Statistical analysis of the data was performed using ANOVA, the Mann-Whitney, Chi-square or Fisher’s exact test (p <  0.05).

**Results:**

There were no significant differences between the relative expression of *CYP19A1* mRNA in the low expression group and the high expression group according to the variables studied. There were no significant differences in *CYP19A1* gene expression in the study and control groups (p = 0.8461). In the relapse group, *CYP19A1* gene expression was significantly higher in the hybrid luminal subtype than in the triple-negative subtype (p = 0.0321), whereas it was significantly lower in HER2-negative cases than in HER2-positive cases (p <  0.0376). Women with locoregional recurrence showed higher expression than women with distant recurrence (p <  0.0001).

**Conclusions:**

The present study found no significant differences between women with high and low expression of the *CYP19A1* gene mRNA or between those in the study group and the control group. However, in women with recurrence, there was increased expression of *CYP19A1* mRNA in those who had the luminal hybrid subtype and locoregional relapse and decreased expression in those negative for HER2.

## Background

Breast cancer is the most common malignancy affecting women worldwide, and in 2018, the global estimate of impacted women was approximately 2,089,000, with a mortality rate of nearly 627,000 [1, 2]. Its incidence is higher in the most developed regions of the world compared to developing and underdeveloped regions [3].

In Brazil, which is a developing country, breast cancer is the second most common malignancy in women after non-melanoma skin cancer, with an estimated 59,700 new cases and 15,403 cases of death from the disease in 2018 [4]. Additionally, approximately 40% of patients who develop disease recurrence die, especially in the first 2 to 3 years, when the risk of recurrence is higher [5–7]. Although physical examination and mammography are important to ensure early diagnosis of the disease and to reduce mortality, breast cancer is still frequently diagnosed in advanced stages in Brazil, resulting in high mortality rates, even with the current therapeutic strategies [8].

It has been suggested that the most appropriate therapeutic and prognostic strategies for breast cancer may be developed using genes that are associated with the development, growth and aggressiveness of breast cancer as biomarkers [9, 10]. This includes the *CYP19A1* gene that encodes the aromatase enzyme, which is involved in estrogen biosynthesis, as it promotes androgen aromatization in estrogens [11, 12]. The *CYP19A1* gene has been studied as a prognostic marker of breast cancer due to its genetic control in estrogen biosynthesis [13, 14]. This gene has tissue-specific promoters, and principally, normal breast adipose tissue maintains low levels of aromatase expression primarily via the I.4 distal promoter. However, in breast cancer, an exchange between the I.4 and I.3 promoters and the I.7 and II promoters occurs, leading to increased production of aromatase and local estrogen [15, 16].

Some studies have examined *CYP19A1* gene expression in breast cancer using quantitative reverse transcription polymerase chain reaction (RT-PCR), which is considered a standard method for the quantitative measurement of gene expression; however, many of these studies have shown controversial results [16, 17]. Miyoshi et al. found no significant association between *CYP19A1* expression levels and breast cancer [18]. On the other hand, Friesenhengst et al. evaluated *CYP19A1* expression in tumors of women with breast cancer, and the results showed a significant association between high *CYP19A1* gene expression and estrogen receptor expression, menopausal status, metastasis-free survival, overall survival, disease-free survival and local and distant recurrence [11]. Thus, the controversies surrounding the gene expression of *CYP19A1* in breast tumor studies and, to the best of our knowledge, the absence of studies analyzing the peripheral blood of women with recurrent breast cancer led to the design of this study.

## Methods

### Patients

This cross-sectional study involved 146 women from 34 to 80 years of age who had breast cancer and received care at the Mastology Clinic of Perola Byington Hospital (Sao Paulo, Brazil) between July and September 2018. The Internal Review Board of the Federal University of Piauı and Perola Byington Hospital approved the study under number CAAE: 43447015.8.0000, and all the patients signed an informed consent form prior to admission. The women were first divided into two groups, with low and high expression of *CYP19A1* with 73 patients each. Subsequently, women were divided into two groups, without recurrence (control, *n* = 85) and with recurrence (study, *n* = 61). Women who were over 18 years of age, with and without breast cancer recurrence in the operable stage, who were diagnosed and treated in the past 10 years and had histologically confirmed diagnoses (disease at diagnosis) were included in the study. Women with a history of another neoplasm, a serious concomitant disease or an initial diagnosis of metastatic breast cancer were excluded from the study.

### Blood sampling

Peripheral blood was collected by a specialized technician using a disposable syringe and needle after medical consultation. The first 1 mL of peripheral blood was discarded to prevent contamination by epidermal cells. A 1 mL sample of total peripheral blood from each patient was preserved in 3 mL TRIzol (Invitrogen; Thermo Fisher Scientific, Inc.) and stored at − 80 °C until RNA extraction.

### Total RNA extraction and cDNA synthesis

RNA extraction was performed using TRIzol reagent (Invitrogen; Thermo Fisher Scientific, Inc.) according to the manufacturer’s instructions. RNA concentration, integrity and purity were analyzed using a NanoDrop 1000 spectrophotometer (Thermo Fisher Scientific, Inc.) and agarose gel electrophoresis. Complementary DNA (cDNA) was synthesized from 2000 ng RNA using SuperScript III First-Strand Synthesis System (Invitrogen; Thermo Fisher Scientific, Inc.) with a total reaction volume of 20 μL containing 50 μM Oligo (dT) 20, 10 mM DNTP, 1 mL 10X RT buffer, 0.1 M DTT, 40 U/μL RNaseOUT and 200 U/μl SuperScript III RT. The incubation conditions for reverse transcription (RT) were 50 °C for 60 min and 70 °C for 15 min. The samples were placed in long-term storage at 4 °C. The cDNA was kept at − 20 °C and was diluted 10-fold prior to use in the quantitative RT-PCR.

### Quantitative RT-PCR

*CYP19A1* mRNA expression was determined by quantitative RT-PCR using Power SYBR Green PCR Master Mix (Applied Biosystems; Thermo Fisher Scientific, Inc.) and an ABI 7500 detection system equipped with SDS v1.4 software. The following primers were used for detection and quantitation of *CYP19A1* mRNA: sense primer, 5′-CACATCCTCAATACCAGGTCC-3′; antisense primer, 5′-CAGAGATCCAGACTCGCATG-3′. *BETA-ACTIN* (*ACTB*) was used as an endogenous normalization control. The following primers were used for *ACTB*: sense primer, 5′-CACTGTGTTGGCGTACAGGT-3′ and antisense primer, 5′-AAATCTGGCACCACACCTTC-3′. Reactions were performed in a final volume of 13 μL, containing 3 μL DNA sample, 6.4 μL SYBR Green Master Mix (Applied Biosystems; Thermo Fisher Scientific, Inc.), 0.4 μL primers (Custom TaqMan Gene Expression Assays, Applied Biosystems; Thermo Fisher Scientific, Inc.), and 2.9 μL of ultrapure sterile water, in 96-well plates using the StepOne Real-Time PCR System (Applied Biosystems, Foster City, CA, USA). After initial denaturation for 10 min at 95 °C, the samples were subjected to 40 amplification cycles, consisting of two steps: 15 s at 95 °C and 1 min at 60 °C. Samples were evaluated in duplicate, and two negative controls were added to each plate containing the same reaction compounds, but the DNA sample was replaced with water. Relative quantitation of *CYP19A1* mRNA expression as a target was performed using the 2 ^ -ΔCT method using the mean values obtained from the threshold cycle (CT) of 146 samples and the *ACTB* CT values as an endogenous control.

### Statistical analysis

To evaluate the associations between *CYP19A1* expression and clinical and histopathologic variables, the Mann-Whitney, Chi-square or Fisher’s exact test and unidirectional ANOVA with multiple comparisons were performed using the Bonferroni post-test method. The values of p <  0.05 were interpreted as statistically significant. All statistical analyses were performed with GraphPad Prism software 6.0 (GraphPad Software, San Diego, CA, USA).

## Results

### Correlations between *CYP19A1* mRNA levels and histopathologic features

Using the median as the cut-off point, the patients were divided in low expression group and high expression group of *CYP19A1*. There were no significant differences between the relative expression of *CYP19A1* mRNA in the low expression group and the high expression group according to the variables studied (Table 1). Patients were classified according to their sensitivity to endocrino therapy (ET) based on 2nd international consensus guidelines for advanced breast cancer, developed by European School of Oncology and European Society of Medical Oncology [19], responsive to ET, when relapses occur after 2 years of adjuvant ET or resistant when a relapses occurs in the first 2 years of adjuvant ET. In the current study, 44.27% of patients were considered responsive to endocrine therapy and 55.73% considered unresponsive.

**Table 1:** Correlations between the levels of *CYP19A1* mRNA and histopathologic features in women with breast cancer

Variables Median age, 54 y (range, 34–82 y)	N (%)	***CYP19A1*** Expression		***p*** value
		Low (%)	High (%)	
**Age**				
≤50 years	60 (41.1)	31 (21.2)	29 (19.9)	0.73
> 51 years	86 (58.9)	42 (28.8)	44 (30.1)	
**Tobacco use**				
Yes	55 (37.7)	30 (20.5)	25 (17.1)	0.39
No	91 (62.3)	43 (29.5)	48 (32.9)	
**Menopausal status**				
Premenopausal	63 (43.2)	31 (21.2)	32 (21.9)	0.86
Postmenopausal	83 (56.8)	42 (28.8)	41 (28.1)	
**Tumor Grade**				
G1	14 (9.6)	3 (2.1)	11 (7.5)	0.07
G2	107 (73.3)	57 (39.0)	50 (34.2)	
G3	25 (17.1)	13 (8.9)	12 (8.2)	
**N Classification**				
N0-N1	127 (87.0)	62 (42.5)	65 (44.5)	0.46
N2-N3	19 (13.0)	11 (7.5)	8 (5.5)	
**Tumor stage**				
I	19 (13.0)	7 (4.8)	12 (8.2)	0.40
II	74 (50.7)	40 (27.4)	34 (23.3)	
III	53 (36.3)	26 (17.8)	27 (18.5)	
**Molecular subtype**				
Luminal A	16 (11.0)	6 (4.1)	10 (6.8)	0.06
Luminal B	68 (45.2)	36 (24.7)	32 (21.9)	
Her2 overexpression	23 (15.8)	12 (8.2)	11 (7.5)	
Triple-negative	31 (21.2)	19 (13.0)	12 (8.2)	
Hybrid Luminal	8 (6.8)	8 (5.5)	0 (0.0)	
**Estrogen receptors**				
Positive Negative	105 (71.9) 41 (28.1)	52 (35.6) 21 (14.4)	53 (36.3) 20 (13.7)	0.85
**Progesterone receptors**				
Positive Negative	97 (66.4) 49 (33.6)	49 (33.6) 24 (16.4)	48 (32.9) 25 (17.1)	0.86
**HER2**				
Positive Negative	35 (24.0) 111 (76.0)	14 (9.6) 59 (40.4)	21 (14.4) 52 (35.6)	0.17
**Histological type**				
Ductal	104 (71.2)	50 (34.2)	54 (37.0)	0.73
Lobular	8 (5.5)	4 (2.7)	4 (2.7)	
Other	34 (23.3)	19 (13.0)	15 (10.3)	
**Recurrence**				
Yes	61(41.8)	32 (21.9)	29 (19.9)	0.61
No	85 (58.2)	41(28.1)	44 (30.1)	

### Association of relative expression of *CYP19A1* mRNA with the clinical and histopathologic features

**Fig. 1:**
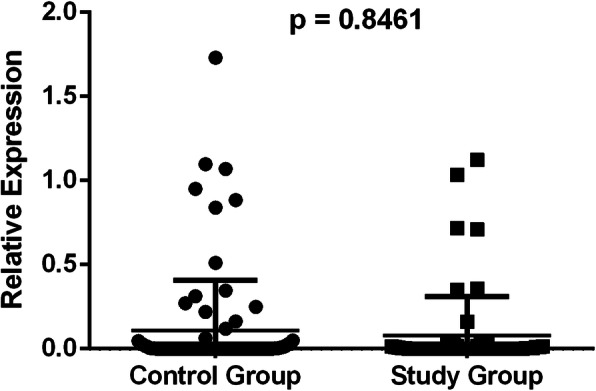
Relative expression of *CYP19A1* mRNA in the study group compared to that in the control group

**Fig. 2:**
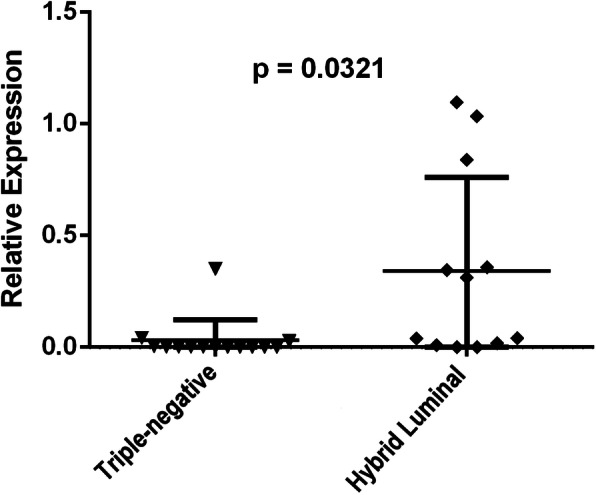
Relative expression of *CYP19A1* mRNA in the relapse group of women who had the hybrid luminal molecular subtype compared to that in those with the triple negative subtype

There were no significant differences in the relative expression of *CYP19A1* mRNA in the study group compared to the control, p = 0.8461 (Fig. 1). In the study group, *CYP19A1* mRNA expression was significantly higher in patients with a hybrid luminal molecular subtype than in patients with a triple-negative subtype, p = 0.0321 (Fig. 2). There were no significant differences in the relative expression of *CYP19A1* mRNA in women with locoregional recurrence in the pre or post menopause (p = 0.116). No other associations were observed between the relative expression of *CYP19A1* mRNA and the other variables studied, such as age, use of tobacco, menopausal status, grade, nodal status (N), tumor stage, estrogen and progesterone receptor, HER2 and histological type.

### Correlations between the median expression levels of *CYP19A1* mRNA and histopathologic features according to recurrence

Using the median as the cut-off point, the median expression of *CYP19A1* mRNA was classified as high or low and then analyzed to determine its association with clinical and histopathological features (Table 2). The group of women with recurrence of breast cancer and negative HER2 receptor expression showed reduced *CYP19A1* mRNA levels compared to those with positive HER2 receptor expression (p < 0.0376). Further, the mRNA expression of *CYP19A1* was significantly higher in women with locoregional recurrence than in women with distant recurrence (p < 0.0001). There was no significant difference between the expression of *CYP19A1* and the other variables studied.

**Table 2:** Correlations between the median expression levels of *CYP19A1* mRNA and histopathologic features according to relapse

Variables	Relapse (***n*** = 61)		***P*** values	Nonrelapsed (***n*** =85)		
	Low	High		Low	High	***P*** values
	n (%)	n (%)		n (%)	n (%)	
**Age**						
≤50 years	13 (21.3)	12 (19.7)	0.877	19 (22.4)	16 (18.8)	0.568
> 51 years	18 (29.5)	18 (29.5)		24 (28.2)	26 (30.6)	
**Tobacco use**						
Yes	13 (21.3)	10 (16.4)	0.488	18 (21.2)	14 (16.5)	0.327
No	18 (29.5)	20 (32,8)		24 (28.2)	29 (34.1)	
**Menopausal status**						
Premenopausal	12 (19.7)	14 (23.0)	0.529	20 (23.5)	17 (20.0)	0.574
Postmenopausal	19 (31.1)	16 (26.2)		23 (27.1)	25 (29.4)	
**Tumor Grade**						
G1	0 (0.0)	4 (6.6)	0.109	3 (3.5)	7 (8.2)	0.395
G2	26 (42.6)	22 (36.1)		30 (35.3)	29 (34.1)	
G3	5 (8.2)	4 (6.6)		9 (10.6)	7 (8.2)	
**N Classification**						
N0-N1	27 (44.3)	24 (39.3)	0.454	36 (42.4)	40 (47.1)	0.273
N2-N3	4 (6.6)	6 (9.8)		6 (7.1)	3 (3.5)	
**Tumor stage**						
I	3 (4.9)	3 (4.9)	0.216	4 (4.7)	9 (10.6)	0.247
II	19 (31.1)	12 (19.7)		21 (24.7)	22 (25.9)	
III	9 (14.8)	15 (24.6)		17 (20.0)	12 (14.1)	
**Molecular subtype**						
Luminal A	1 (1.6)	2 (3.3)	0.4455	4 (4.7)	8 (9.4)	0.182
Luminal B	17 (27.9)	18 (29.5)		19 (22.4)	22 (25.9)	
Her2 overexpression	4 (6.6)	6 (9.8)		8 (9.4)	5 (5.9)	
Triple-negative	9 (14.8)	4 (6.6)		10 (11.8)	8 (9.4)	
**Estrogen receptors**						
Positive	22 (36.1)	25 (41.0)	0.250	30 (35.3)	18 (21.2)	0.532
Negative	9 (14.8)	5 (8.2)		12 (14.1)	15 (17.6)	
**Progesterone receptors**						
Positive Negative	22 (36.1) 9 (14.8)	19 (31.1) 11 (18.0)	0.525	27 (31.8) 15 (17.6)	29 (34.1) 14 (16.5)	0.759
**HER2**						
Positive Negative	5 (8.2) 26 (42.6)	12 (19.7) 18 (29.5)	**< 0.0376**	9 (10.6) 33 (38.8)	9 (10.6) 34 (40.0)	0.955
**Histological type**						
Ductal	23 (37.7)	23 (37.7)	0.584	26 (30.6)	32 (37.6)	0.154
Lobular	3 (4.9)	1 (1.6)		1 (1.2)	3 (3.5)	
Other	5 (8.2)	6 (9.8)		15 (17.6)	8 (9.4)	
**Recurrence type**						
Locoregional	0 (0.0)	24 (39.3)	**< 0.0001**	–	–	–
Distant metastasis	19 (31.1)	18 (29.5)		–	–	

## Discussion

The detection of circulating *CYP19A1* mRNA by RT-PCR has been shown to be significantly increased in breast cancer compared to normal controls, and quantitative RT-PCR is considered a sensitive and reliable method for studying mRNA in a variety of sites, such as bone marrow, lymph nodes, tissues and blood [20–23]. However, according to a survey of the literature, the evaluation of *CYP19A1* mRNA by quantitative RT-PCR in the total blood of women with breast cancer recurrence was not reported, but some studies solely involving normal, peritumoral and tumoral tissues are available [11, 18]. Peripheral blood has been used as a clinical sample for gene expression analysis in breast cancer, since peripheral blood samples are readily available, their acquisition is minimally invasive and they can be collected at low cost, thus making them an attractive alternative modality for diagnostic and prognostic purposes in cancer research [24, 25]. According to some authors, there is a correlation between *CYP19A1* mRNA levels in peripheral blood leukocytes and target tissues [26, 27].

In the present study, comparison of peripheral blood *CYP19A1* gene expression levels by quantitative RT-PCR between women with low and high expression and women with nonrelapsed breast cancer (control) and women with relapsed cancer (study) showed no statistically significant differences. In the group with relapsed cancer, *CYP19A1* gene expression was significantly higher in women with a hybrid luminal molecular subtype than in women with a triple-negative subtype. Regarding tumor characteristics, the group of women with breast cancer recurrence showed a significant reduction in *CYP19A1* mRNA in women with HER2-negative tumors compared to those with HER2-positive tumors. Additionally, *CYP19A1* mRNA expression was significantly higher in women with locoregional recurrence than in those with distant recurrence, and there was no difference in relation to the other variables studied.

On evaluating the expression of the *CYP19A1* gene in breast cancer tissue, Friesenhengst et al. [11] detected an association between the high expression of *CYP19A1* in breast tumors and the incidence of breast cancer recurrence. However, consistent with our results, Girault et al. [28] and Licznerska et al. [29] evaluated *CYP19A1* gene expression in breast cancer tissue and found no associations between *CYP19A1* mRNA levels and disease recurrence. Darlix et al. [30] showed longer survival in women with hybrid luminal breast cancer than in those with triple-negative breast cancer, while other authors, such as Friesenhengst et al. [11] and Brown et al. [31], showed no association between *CYP19A1* mRNA expression levels and molecular subtypes of breast cancer. However, these studies were performed in tumor tissue and had a smaller sample size than the present study.

Some authors have not shown an association between *CYP19A1* gene expression in women with breast cancer and HER2 receptor status [11, 32, 33]. However, findings similar to those in this study were found by Subbaramaiah et al. [34], who showed lower levels of aromatase enzyme and *CYP19A1* activity in HER2-negative tumors than in HER2-positive tumors. Some authors have shown that HER2 overexpression is the main determinant of increased expression of cyclooxygenase-2 and synthesis of prostaglandin E2 in breast tumor cells, which in turn, leads to increased *CYP19A1* gene expression and aromatase activity [34–36].

Bollet et al. [32] showed a significant association between low expression of the *CYP19A1* gene and an increased risk of locoregional recurrence. On the other hand, other studies have shown an association between the complete absence of *CYP19A1* gene expression and shorter relapse-free survival [29, 37]. Furthermore, consistent with our results, Friesenhengst et al. [11] and Salhab et al. [33] reported that high expression of the *CYP19A1* gene was associated with increased locoregional recurrence. Estrogen synthesis in situ is believed to be primarily catalyzed by the enzyme aromatase, which is often overexpressed in breast tumors, thus explaining the increased levels of *CYP19A1* mRNA in patients with locoregional breast recurrence compared with those in patients with recurrence in regions more distal to the tumor such as liver, brain and bones [15, 38].

Friesenhengst et al. [11] showed that *CYP19A1* mRNA levels were significantly elevated in postmenopausal breast cancer patients. Tüzüner et al. [39] showed that the expression levels of the *CYP19A1* gene were significantly decreased in patients older than 50 years. However, in agreement with our results, many studies have not shown any association between *CYP19A1* gene expression and variables such as age, tobacco use, menopausal status, grade, nodal status, tumor stage, estrogen receptor, progesterone and histological type [18, 28, 29, 32, 37].

## Conclusion

The present study found no significant differences between women with high and low expression of the *CYP19A1* gene mRNA or between those in the study and control groups. However, in women with recurrence, there was increased expression of *CYP19A1* mRNA in those who had the luminal hybrid subtype and locoregional relapse and decreased expression in those negative for HER2; nevertheless, further studies should be performed to consolidate the findings of the present study.

## Data Availability

The datasets used and/or analysed during the current study are available from the corresponding author on reasonable request.

## Abbreviations

ACTB: Beta-actin
cDNA: Complementary DNA
CT: Threshold cycle
N: Nodal status
RT: Reverse transcription
RT-PCR: Reverse transcription polymerase chain reaction

## References

[1] Bray F, Ferlay J, Soerjomataram I, Siegel RL, Torre LA, Jemal A (2018). Global cancer statistics 2018: GLOBOCAN estimates of incidence and mortality worldwide for 36 cancers in 185 countries. CA Cancer J Clin.

[2] Ferlay J, Colombet M, Soerjomataram I, Mathers C, Parkin DM, Piñeros M (2019). Estimating the global cancer incidence and mortality in 2018: GLOBOCAN sources and methods. Int J Cancer.

[3] Ghoncheh M, Pournamdar Z, Salehiniya H (2016). Incidence and mortality and epidemiology of breast Cancer in the world. Asian Pac J Cancer Prev.

[4] Quintanilha LF, Souza LN, Sanches D, Demarco RS, Fukutani KF (2019). The impact of cancer campaigns in Brazil: a Google trends analysis. Ecancermedicalscience.

[5] Jemal A, Thun MJ, Ries LA, Howe HL, Weir HK, Center MM (2008). Annual report to the nation on the status of cancer, 1975-2005, featuring trends in lung cancer, tobacco use, and tobacco control. J Natl Cancer Inst.

[6] Gerber B, Freund M, Reimer T (2010). Recurrent breast cancer: treatment strategies for maintaining and prolonging good quality of life. Dtsch Arztebl Int.

[7] Voinea SC, Sandru A, Blidaru A (2017). Management of Breast Cancer Locoregional Recurrence. Chirurgia (Bucur).

[8] Anderson KN, Schwab RB, Martinez ME (2014). Reproductive risk factors and breast cancer subtypes: a review of the literature. Breast Cancer Res Treat.

[9] Costa-Silva DR, da Conceição B-OM, Borges RS, Campos-Verdes LM, da Silva-Sampaio JP, Escorcio-Dourado CS (2017). Insulin-like growth factor 1 gene polymorphism in women with breast cancer. Med Oncol.

[10] Campos-Verdes LM, da Silva-Sampaio JP, Costa-Silva DR, de Oliveira VA, Junior AMC, Silva VC (2018). Genetic polymorphism of calcium-sensing receptor in women with breast cancer. Med Oncol.

[11] Friesenhengst A, Pribitzer-Winner T, Miedl H, Pröstling K, Schreiber M (2018). Elevated aromatase (CYP19A1) expression is associated with a poor survival of patients with estrogen receptor positive breast Cancer. Horm Cancer.

[12] Savolainen-Peltonen VV, Wang F, Turpeinen U, Hämäläinen E, Haanpää M (2018). Estrogen biosynthesis in breast adipose tissue during menstrual cycle in women with and without breast cancer. Gynecol Endocrinol.

[13] Simpson E, Santen RJ (2015). Celebrating 75 years of oestradiol. J Mol Endocrinol.

[14] Zhao H, Zhou L, Shangguan AJ, Bulun SE (2016). Aromatase expression and regulation in breast and endometrial cancer. J Mol Endocrinol.

[15] Bulun SE, Lin Z, Imir G, Amin S, Demura M, Yilmaz B (2005). Regulation of aromatase expression in estrogen-responsive breast and uterine disease: from bench to treatment. Pharmacol Rev.

[16] Amatori S, Persico G, Fanelli M (2017). Real-time quantitative PCR array to study drug-induced changes of gene expression in tumor cell lines. J Cancer Metastasis Treat.

[17] El Hadi Hicham, Abdellaoui-Maane Imane, Kottwitz Denise, El Amrani Manal, Bouchoutrouch Nadia, Qmichou Zineb, Karkouri Mehdi, ElAttar Hicham, Errihani Hassan, Fernandez Pedro L, Bakri Youssef, Sefrioui Hassan, Moumen Abdeladim (2017). Development and evaluation of a novel RT-qPCR based test for the quantification of HER2 gene expression in breast cancer. Gene.

[18] Miyoshi Y, Ando A, Hasegawa S, Ishitobi M, Taguchi T, Tamaki Y (2003). High expression of steroid sulfatase mRNA predicts poor prognosis in patients with estrogen receptor-positive breast cancer. Clin Cancer Res.

[19] Cardoso F, Costa A, Norton L, Senkus E, Aapro M, André F (2014). ESO-ESMO 2nd international consensus guidelines for advanced breast Cancer (ABC2). Breast.

[20] Bustin SA, Mueller R (2005). Real-time reverse transcription PCR (qRT-PCR) and its potential use in clinical diagnosis. Clin Sci (Lond).

[21] Wang S, Xu J, Zhang Q (2016). Clinical significance of survivin and vascular endothelial growth factor mRNA detection in the peripheral whole blood of breast cancer patients. Neoplasma.

[22] Gilbey AM, Burnett D, Coleman RE, Holen I (2004). The detection of circulating breast cancer cells in blood. J Clin Pathol.

[23] Yie SM, Luo B, Ye NY, Xie K, Ye SR (2006). Detection of Survivin-expressing circulating cancer cells in the peripheral blood of breast cancer patients by a RT-PCR ELISA. Clin Exp Metastasis.

[24] Sharma P, Sahni NS, Tibshirani R, Skaane P, Urdal P, Berghagen H (2005). Early detection of breast cancer based on gene-expression patterns in peripheral blood cells. Breast Cancer Res.

[25] Aarøe J, Lindahl T, Dumeaux V, Saebø S, Tobin D, Hagen N (2010). Gene expression profiling of peripheral blood cells for early detection of breast cancer. Breast Cancer Res.

[26] Pignatti E, Casarini L, Scaltriti S, Wistuba J, Schlatt S, Rossi A (2012). Aromatase expression in human peripheral blood leucocytes (PBLs) and in various tissues in primates: studies in elderly humans and cynomolgus monkeys. J Med Primatol.

[27] Stratakis CA, Vottero A, Brodie A, Kirschner LS, DeAtkine D, Lu Q (1998). The aromatase excess syndrome is associated with feminization of both sexes and autosomal dominant transmission of aberrant P450 aromatase gene transcription. J Clin Endocrinol Metab.

[28] Girault I, Lerebours F, Tozlu S, Spyratos F, Tubiana-Hulin M, Lidereau R (2002). Real-time reverse transcription PCR assay of CYP19 expression: application to a well-defined series of post-menopausal breast carcinomas. J Steroid Biochem Mol Biol.

[29] Licznerska BE, Wegman PP, Nordenskjold B, Wingren S (2008). In situ levels of oestrogen producing enzymes and its prognostic significance in postmenopausal breast cancer patients. Breast Cancer Res Treat.

[30] Darlix A, Griguolo G, Thezenas S, Kantelhardt E, Thomssen C, Dieci MV (2018). Hormone receptors status: a strong determinant of the kinetics of brain metastases occurrence compared with HER2 status in breast cancer. J Neuro-Oncol.

[31] Brown KA, Iyengar NM, Zhou XK, Gucalp A, Subbaramaiah K, Wang H (2017). Menopause is a determinant of breast aromatase expression and its associations with BMI, inflammation, and systemic markers. J Clin Endocrinol Metab.

[32] Bollet MA, Savignoni A, De Koning L, Tran-Perennou C, Barbaroux C, Degeorges A (2009). Tumor aromatase expression as a prognostic factor for local control in young breast cancer patients after breast-conserving treatment. Breast Cancer Res.

[33] Salhab M, Reed MJ, Al Sarakbi W, Jiang WG, Mokbel K (2006). The role of aromatase and 17-beta-hydroxysteroid dehydrogenase type 1 mRNA expression in predicting the clinical outcome of human breast cancer. Breast Cancer Res Treat.

[34] Subbaramaiah K, Howe LR, Port ER, Brogi E, Fishman J, Liu CH (2006). HER-2/neu status is a determinant of mammary aromatase activity in vivo: evidence for a cyclooxygenase-2-dependent mechanism. Cancer Res.

[35] Harris RE, Robertson FM, Abou-Issa HM, Farrar WB, Brueggemeier R (1999). Genetic induction and upregulation of cyclooxygenase (COX) and aromatase (CYP19): an extension of the dietary fat hypothesis of breast cancer. Med Hypotheses.

[36] Brueggemeier RW, Díaz-Cruz ES, Li PK, Sugimoto Y, Lin YC, Shapiro CL (2005). Translational studies on aromatase, cyclooxygenases, and enzyme inhibitors in breast cancer. J Steroid Biochem Mol Biol.

[37] Yoshimura N, Harada N, Bukholm I, Kåresen R, Børresen-Dale AL, Kristensen VN (2004). Intratumoural mRNA expression of genes from the oestradiol metabolic pathway and clinical and histopathological parameters of breast cancer. Breast Cancer Res.

[38] Zhang Z, Yamashita H, Toyama T, Omoto Y, Sugiura H, Hara Y, Wu X, Kobayashi S, Iwase H (2003). Quantitative determination, by real-time reverse transcription polymerase chain reaction, of aromatase mRNA in invasive ductal carcinoma of the breast. Breast Cancer Res.

[39] Tüzüner MB, Öztürk T, Eronat AP, Seyhan F, Kısakesen Hİ, Calay Z (2016). Evaluation of local CYP17A1 and CYP19A1 expression levels as prognostic factors in postmenopausal invasive ductal breast Cancer cases. Biochem Genet.

